# Idiopathic Pubic Symphysis Diastasis in a Patient With Autosomal Dominant Polycystic Kidney Disease: A Rare Case Report

**DOI:** 10.7759/cureus.92657

**Published:** 2025-09-18

**Authors:** Luka Beridze, Lizi Vardiashvili, Zaza Aladashvili, Tika Lekishvili, Tariel Kintsurashvili

**Affiliations:** 1 Internal Medicine, Petre Shotadze Tbilisi Medical Academy, Tbilisi, GEO; 2 Emergency Department, Tbilisi State Medical University - The First University Clinic, Tbilisi, GEO; 3 Medicine, Tbilisi State Medical University, Tbilisi, GEO; 4 Trauma and Orthopaedics, National Center of Surgery, Tbilisi, GEO

**Keywords:** autosomal-dominant polycystic kidney disease, pelvic joint disorders, pelvic x-ray, pubic symphysis diastasis, rare case report, young female patients

## Abstract

Pubic symphysis diastasis (PSD) is characterized by widening of the pubic symphysis, most often associated with pregnancy, childbirth, or trauma, while idiopathic PSD is exceedingly rare. Autosomal dominant polycystic kidney disease (ADPKD) is a multisystem disorder, but PSD has not been previously reported in this context. We present a 22-year-old female with ADPKD and progressive hip discomfort and clicking since childhood. Examination revealed mildly restricted, painful hip motion without instability. Radiography showed a pubic symphysis width of 25.5 mm, with no sacroiliac, traumatic, or degenerative findings. With no obstetric, traumatic, or inflammatory history, idiopathic PSD was diagnosed. The patient was managed conservatively with physical therapy, activity modification, and orthopedic follow-up. This is the first reported case of idiopathic PSD in an ADPKD patient, expanding the spectrum of musculoskeletal manifestations in ADPKD and highlighting the need to consider pelvic structural disorders in unexplained hip or pelvic pain.

## Introduction

Pubic symphysis diastasis (PSD) is defined as a widening of the pubic symphysis greater than 10 mm [1]. It most commonly occurs in association with pregnancy and childbirth, due to hormonal changes and mechanical stress, and can also result from traumatic events such as pelvic fractures or direct pelvic trauma [1]. Idiopathic PSD, in contrast, is extremely rare and is usually identified in childhood or incidentally on imaging, often presenting with subtle symptoms such as hip discomfort, pelvic pain, or gait abnormalities [1]. The rarity of idiopathic PSD and its nonspecific clinical presentation make diagnosis challenging, typically requiring both clinical assessment and radiographic evaluation.

Autosomal dominant polycystic kidney disease (ADPKD) is an inherited, progressive multisystem disorder primarily characterized by renal cyst formation [2]. Cyst growth over time may lead to renal enlargement, hypertension, and chronic kidney disease. Beyond the kidneys, ADPKD can manifest extrarenally, with cystic involvement of the liver and pancreas being most common [2]. Non-cystic manifestations may affect the gastrointestinal tract, vascular system, bones, and cardiac valves [3]. These systemic effects contribute to a wide spectrum of clinical complications, although musculoskeletal involvement-particularly structural pelvic abnormalities not been previously reported. Recognizing potential skeletal manifestations in ADPKD is important for comprehensive patient care and early identification of rare complications.

We report a case of idiopathic PSD in a 22-year-old female with ADPKD. To our knowledge, this is the first documented case of PSD in this context, expanding the spectrum of musculoskeletal manifestations in patients with ADPKD and highlighting the importance of considering pelvic structural disorders in patients presenting with unexplained hip or pelvic pain.

## Case presentation

A 22-year-old female with a diagnosis of autosomal dominant polycystic kidney disease (ADPKD) presented to her healthcare provider with progressive discomfort and a clicking sensation in the hip. She reported that these symptoms had been present since childhood but had gradually intensified over time, affecting her daily activities. She denied any history of trauma, childbirth, or participation in sports or other activities that might predispose her to pubic symphysis injury or overuse. Additional patient characteristics relevant to musculoskeletal assessment, including BMI, skeletal phenotype, and family history, are summarized in Table 1.

**Table 1:** Patient characteristics relevant to musculoskeletal assessment Clinical and anthropometric characteristics of the patient. The table summarizes age, body mass index (BMI), skeletal phenotype, family history, and other relevant risk factors. Findings indicate a 22-year-old female with autosomal dominant polycystic kidney disease (ADPKD), normal BMI, normal skeletal phenotype, no family history of musculoskeletal disorders, and no additional risk factors.

Parameter	Finding	Notes
Age	22 years	Young adult female with ADPKD
BMI	21.8 kg/m²	Within normal range
Skeletal phenotype	Normal build, no evidence of skeletal dysplasia	No abnormal limb proportions, stature within expected range
Family history	Negative for musculoskeletal disorders	Family history positive only for ADPKD
Other risk factors	None reported	No history of trauma, pregnancy, or repetitive overuse activity

On physical examination, hip range of motion was slightly limited and mildly painful, although palpation over the symphysis pubis was non-tender. No pelvic instability was observed, and neurological examination of the lower extremities was unremarkable, with preserved motor strength, reflexes, and sensation.

**Figure 1:**
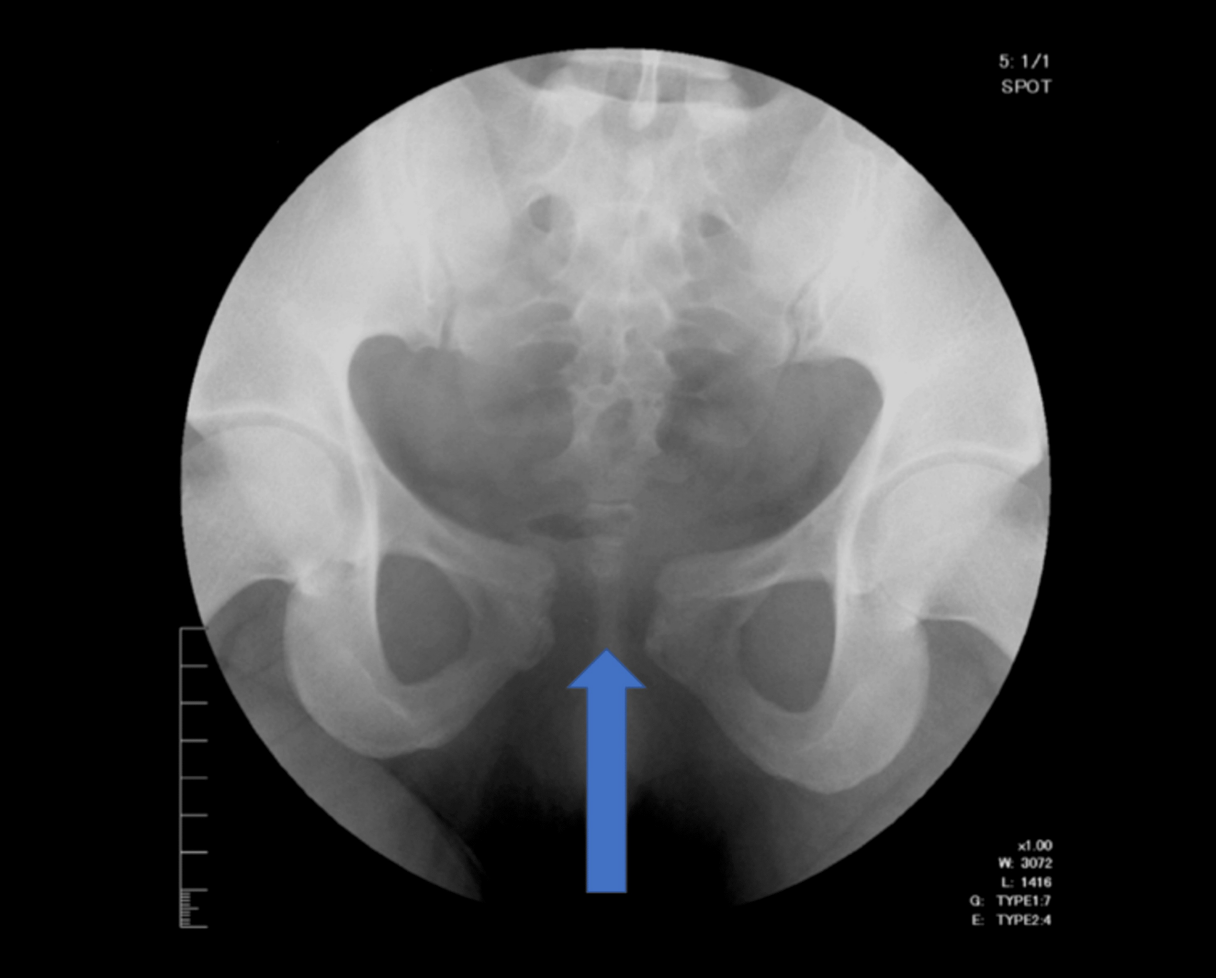
Anteroposterior radiograph of the pelvis showing pubic symphysis diastasis Anteroposterior pelvic radiograph of a 22-year-old female with ADPKD. The arrow indicates widening of the pubic symphysis measuring 25.5 mm. No sacroiliac joint asymmetry, fractures, infection, or degenerative changes are present, consistent with idiopathic pubic symphysis diastasis.

Routine laboratory evaluation revealed stable renal function and no evidence of electrolyte imbalance, consistent with her known ADPKD history. Pelvic radiography demonstrated widening of the symphysis pubis measuring 25.5 mm without sacroiliac joint asymmetry, fractures, signs of infection, or degenerative changes (Figure 1).

Given the absence of trauma, obstetric history, or inflammatory conditions, the findings were most consistent with idiopathic pubic symphysis diastasis. The patient was counseled extensively on conservative management strategies, including activity modification, targeted physical therapy, and regular monitoring. She was referred to orthopedic specialists for ongoing follow-up to assess long-term pelvic stability, guide supportive care, and provide recommendations for symptom management.

## Discussion

The pubic symphysis serves as the firm midline articulation joining the two pubic bones of the pelvis [4]. It consists of the articular surfaces of the pubic bones and an interposed fibrocartilaginous disc, which may contain a central cleft [4]. During activities such as bending, standing, or leg elevation, the joint is exposed to shear stresses that create tensile and sliding forces of varying magnitudes and directions [5]. When walking, the symphysis helps dissipate impact forces transmitted through the pelvic ring. Normally, this joint allows for 1-2 mm of separation during lower limb abduction and permits about 1° of rotation [5].

Pubic symphysis diastasis (PSD) is diagnosed when the gap between the pubic bones exceeds 10 mm [1]. The most frequent causes are pregnancy and childbirth, followed by traumatic injury [1]. Other conditions that may involve the pubic symphysis include congenital anomalies (e.g., bladder exstrophy, cleidocranial dysostosis), inflammatory and infectious osteitis, metabolic disorders (e.g., hyperparathyroidism, hemochromatosis), and, rarely, neoplastic infiltration, usually from metastatic disease [4].

The mechanisms underlying idiopathic PSD are not well defined. In our case, the absence of trauma, pregnancy, inflammation, or repetitive stress supported an idiopathic diagnosis. The patient also had autosomal dominant polycystic kidney disease (ADPKD), a progressive multisystem disorder characterized by bilateral renal cysts, renal enlargement, and involvement of other organs such as the liver, pancreas, spleen, heart, and meninges [2]. ADPKD arises from mutations in PKD1 on chromosome 16p13.3 (about 85% of cases) or PKD2 on chromosome 4q21 (about 15%) [2]. PKD1 encodes polycystin-1 (PC1), a membrane protein with a large extracellular domain, 11 transmembrane segments, and a short intracellular tail. PC1 localizes to focal adhesions, cilia, tight and adherens junctions, and desmosomes, where it is essential for intercellular and cell-matrix signaling [2].

Recently, interest has grown regarding bone involvement in ADPKD. Polycystins and primary cilia in osteoblasts and osteocytes suggest their role in bone regulation. PC1 acts as a mechanosensor, influencing osteoblast gene activity and differentiation. Dysfunction of PKD1 in bone tissue may contribute to abnormal skeletal growth, decreased cortical thickness, lower bone mineral density, and osteopenia [3,6-9]. PC1 also regulates chondrocyte and osteoblast differentiation as well as adipogenesis, with missense mutations delaying bone formation. Animal studies show that loss of PC1 reduces mineralized bone in calvaria and long bones, disrupting both intramembranous and endochondral ossification, and leading to reduced density due to impaired bone formation rather than enhanced resorption [3,6,7,10].

In contrast, skeletal effects in human ADPKD tend to be mild or undetectable compared with experimental models [3]. This may reflect species differences or the limited sensitivity of current clinical methods. Still, disruption of PKD1 provides a plausible mechanistic link to musculoskeletal abnormalities such as PSD.

In our patient, the coexistence of idiopathic PSD with ADPKD raises the possibility that connective tissue and skeletal abnormalities may represent underrecognized extrarenal manifestations of the disease. Given the expression of polycystins in bone and cartilage, their dysfunction could predispose to ligamentous laxity, abnormal joint mechanics, or reduced bone density, increasing susceptibility to pelvic instability even in the absence of obstetric, traumatic, or inflammatory triggers. To contextualize the rarity and characteristics of our case, Table 2 compares the pubic symphysis diastasis (PSD) and relevant clinical features of our patient with previously reported cases. This comparison highlights the unusually large PSD in our patient relative to postpartum and idiopathic cases reported in the literature.

**Table 2:** Comparison of pubic symphysis diastasis across reported cases Summarizes pubic symphysis diastasis (PSD) measurements and key clinical features of the current ADPKD patient compared with previously reported cases. The table highlights the unusually large PSD in our patient, emphasizing the rarity and clinical significance of this finding in the context of ADPKD.

Study / Authors	Age (years)	Pubic Symphysis Diastasis (PSD)	Key Findings / Notes
Current Case	22	25.5 mm	Female with ADPKD; normal skeletal phenotype; no other musculoskeletal disorders
Chaudhary et al. [11]	24	16 mm	G5P2A2L1; postpartum PSD; severe hip pain, difficulty walking; no comorbidities
Chavla et al. [12]	22	15 mm	Abnormal widening of pubic symphysis; sacroiliac joints normal
Chavla et al. [12]	Not specified	16 mm	G4P2A1; postpartum PSD with ligament disruption; loculated collection near symphysis

## Conclusions

This case highlights the first reported occurrence of idiopathic pubic symphysis diastasis in a patient with autosomal dominant polycystic kidney disease, expanding the spectrum of potential extrarenal manifestations. Recognition of this association is clinically important, as it emphasizes the need to consider structural pelvic disorders in ADPKD patients presenting with unexplained hip or pelvic discomfort and supports the use of imaging to guide timely diagnosis and management. While a single case cannot establish causality, the biologic plausibility of polycystin-related skeletal alterations suggests that connective tissue or bone abnormalities may contribute to joint instability in this population. Further clinical and basic science studies are warranted to clarify this relationship and to determine whether musculoskeletal involvement should be recognized as part of the systemic manifestations of ADPKD.
